# *Streptococcus pneumoniae* Endopeptidase O (PepO) Elicits a Strong Innate Immune Response in Mice via TLR2 and TLR4 Signaling Pathways

**DOI:** 10.3389/fcimb.2016.00023

**Published:** 2016-02-29

**Authors:** Hong Zhang, Lihua Kang, Hua Yao, Yujuan He, Xiaofang Wang, Wenchun Xu, Zhixin Song, Yibing Yin, Xuemei Zhang

**Affiliations:** Department of Laboratory Medicine, Key Laboratory of Diagnostic Medicine (Ministry of Education), Chongqing Medical UniversityChongqing, China

**Keywords:** *Streptococcus pneumonia*, PepO, innate immunity, TLR2, TLR4, signaling pathway

## Abstract

Interaction between virulence factors of *Streptococcus pneumoniae* and innate immune receptors elicits host responses through specific signaling pathways during infection. Insights into the signaling events may provide a better knowledge of the starting events for host-pathogen interaction. Here we demonstrated a significant induction of innate immune response elicited by recombinant *S. pneumoniae* endopeptidase O (rPepO), a newer pneumococcal virulence protein, both *in vivo* and *in vitro*. Intratracheal instillation of rPepO protein resulted in significant increase of cytokines production and neutrophils infiltration in mouse lungs. TLR2 or TLR4 deficient mice subjected to rPepO treatment showed decreased cytokines production, reduced neutrophils infiltration and intensified tissue injury as compared with WT mice. Upon stimulation, cytokines TNF-α, IL-6, CXCL1, and CXCL10 were produced by peritoneal exudate macrophages (PEMs) in a TLR2 and TLR4 dependent manner. rPepO-induced cytokines production was markedly decreased in TLR2 or TLR4 deficient PEMs. Further study revealed that cytokines induction relied on the rapid phosphorylation of p38, Akt and p65, not the activation of ERK or JNK. While in TLR2 or TLR4 deficient PEMs the activation of p65 was undetectable. Taken together, these results indicate for the first time that the newer pneumococcal virulence protein PepO activates host innate immune response partially through TLR2 and TLR4 signaling pathways.

## Introduction

*Streptococcus pneumoniae*, a gram positive bacterium, asymptomatically colonizes the upper respiratory airway and causes common clinical syndromes such as otitis media, sinusitis, bronchitis, and empyema or even severe life-threatening diseases including pneumonia, meningitis, and septicemia (van Der Poll and Opal, 2009; Cole et al., 2014). *S. pneumoniae* infections have led to significant morbidity and mortality worldwide especially in children under 5 years old and the elder over 65 years old in developing countries (O'Brien et al., 2009). Main approaches to prevent and treat diseases caused by *S. pneumoniae* are using effective vaccines and antibiotics. However, what challenge us most is the fast development of antibiotic-resistant *S. pneumoniae* strains and the existence of a number of problems with the application of vaccines. Pneumococcal polysaccharide vaccine and capsular polysaccharide (CPS)–protein conjugate vaccine (PCV) protect people against serotypes but give rise to the risk for natural serotype switching (Kadioglu et al., 2008; Goncalves et al., 2014). Protein-based vaccine could avoid the problem of poor polysaccharide immunogenicity among infants and elderly people and would probably protect host from most pneumococcal strains (Kadioglu et al., 2008; Liu et al., 2014). Therefore, it is worthwhile for researchers to discover novel virulence factors and to illustrate the interaction between virulence factors and host, which may contribute to the development of anti-bacterial drugs and may broaden the scope of researches on protein vaccines.

*S. pneumoniae* endopeptidase O (PepO) is a novel discovered 72KD protein and a predicted metallo-endopeptidase sharing homology with M13 peptidase family. Mammalian endopeptidases, a member of M13 peptidase family, involve in regulation of many physiological and pathological progress including various aspects of immune response. In the content of pneumococcal PepO, Agarwal and colleagues have proved that PepO promotes *S. pneumoniae* evade host innate immunity and invade host cells through binding to plasminogen and fibronectin (Agarwal et al., 2013). They further demonstrated that PepO binds to complement C1q, modulating the attack by complement and promoting adherence to host cell (Agarwal et al., 2014). A PepO-deficient strain showed decreased ability to adherent to and invade host cells compared with its wild-type strain. Thus, involvement of PepO in virulence has been well illustrated. Whether and how PepO activates Toll-like receptors (TLRs) or other pattern recognition receptors (PRRs) are still not known. Research on the effect of PepO on host immune response will provide us a better knowledge of the starting events for host-pathogen interaction.

The innate immune response builds up the first line of defense response against bacterial invasion. As important innate immune cells, macrophages respond immediately to diverse microbial pathogens and quickly control the replication of the invading pathogen (Medzhitov and Janeway, 2000; Hato and Dagher, 2014; Elia et al., 2015). Microbial components could initiate the innate immune response through ligating to PRRs such as NOD-like receptors and TLRs (Politis and Vogel, 2005; Brekke et al., 2007; Groves et al., 2008). Recognition of peptidoglycan from Gram-positive bacteria by TLR2 or lipopolysaccharide (LPS) from Gram-negative bacteria by TLR4 is universally accepted. The binding of ligands to TLRs could trigger signal cascade resulting in secretion of inflammatory cytokines that are mediated by phosphorylation of MAPKs, IFN regulatory factors, and NF-κB (Medzhitov, 2007). Three types of MAPKs- ERK, JNK, and p38 are activated independently or simultaneously in mammalian cells. Phosphatidylinositol 3-kinases (PI3Ks) can be activated during TLR signaling through direct interaction with the receptor or adaptor molecular TIRAP or MyD88, which leads to the phosphorylation of its downstream target, Akt, and the activation of NF-κB (Akira and Takeda, 2004; Santos-Sierra et al., 2009).

It is demonstrated that the pneumococcal virulent proteins directly participate in pathogenesis through interacting with the host innate immune system (Kawai and Akira, 2011; Dong et al., 2014). For example, pore-forming cytolysin pneumolysin (Ply) is known as a major virulence determinant of *S. pneumoniae*, conferring protection from complement-mediated clearance and promoting *S. pneumoniae* crossing through the epithelial barrier and establishing invasive infections (Yuste et al., 2005; Gauthier et al., 2007; Marini et al., 2014). In contrast, this key virulence protein can become the focus of host innate immune system, inducing inflammatory and apoptotic events via TLR4, which optimizes pathogen clearance (Srivastava et al., 2005; Shoma et al., 2008; Bewley et al., 2014). In addition, other pneumococcal virulence proteins such as adherence and virulence factor A (PavA), RrgA Pneumococcal Pilus Type 1 Protein and glycosyl hydrolase 25 participating in invasion protein (GHIP) have been reported to be essential factors for bacterial virulence and induction of optimal cytokines production by innate immune cells (Noske et al., 2009; Basset et al., 2013; Dong et al., 2014). Despite these advances, research on the innate immune response induced by pneumococcal virulence proteins and the related molecular mechanisms is still limited. Further clarification is needed, particularly for these new virulence factors.

In the present research, we have explored the roles of rPepO in activation of innate immunity, as well as the involved molecular mechanism.

## Materials and methods

### Reagents

*Escherichia coli* K12- derived ultra-purified LPS was obtained from InvivoGen (San Diego, CA). Anti-PepO antibody was from mice immunized with rPepO. Mouse anti-actin monoclonal antibody was purchased from Santa Cruz Biotechnology (Santa Cruz, CA). Rabbit anti- phospho- MAPKs, anti-phospho- P65, anti- phospho- Akt, anti-MAPKs, anti-Akt, and anti-lamin B monoclonal antibodies were bought from Cell Signaling Technology (Beverly, MA). P38 inhibitor SB203580, ERK inhibitor U0126, JNK inhibitor SP600125, IκB-α phosphorylation inhibitor BAY11-7082, Janus kinase inhibitor AG490, and PI3K inhibitor LY294002 were purchased from Cell Signaling Technology. AG490, SP600125, PD98059, and BAY117082 were dissolved in DMSO, while LY294002 and SB203580 were dissolved in endotoxin-free water.

### Preparation of recombinant protein PepO

The details were as Vaibhav Agarwal and colleagues described earlier (Agarwal et al., 2013). Briefly, the full-length *pepO* gene was obtained by PCR from D39 chromosomal DNA with primers 5-CCATGGCACGTTATCAGATGATTT-3 and 5-CTCGAGCCAAATAATCACGCGCTC-3, which incorporated flanking NcoI and XhoI (underlined) restriction sites. The amplified DNA was cloned in pJET1.2 (Fermentas) and later into pET28a (Novagen) for protein expression, after which pneumococcal PepO with an N-terminal His_6_ tag was expressed in *E. coli* BL21 (DE3) (Stratagene) and purified using a nickel-nitrilotriacetic acid column (GE healthcare) according to the manufacture's instruction. Then endotoxin-free plastic ware, glassware, and water were used. To remove lipopolysaccharide (LPS) from rPepO preparation as much as possible, protein fractions were incubated with polymyxin B (PmB)—agarose (Sigma, Santa Clara, CA) with end-over-end rotation at 4°C overnight. Residual LPS in the rPepO preparation was measured by the Limulusamoebocyte lysate (LAL) assay (Lonza), following the manufacturer's instructions. rPepO purity was determined using Coomassie Blue Staining. Aliquots were stored at −80°C until they were used. To exclude the effects of contaminated LPS, the rPepO preparation was incubated with 100 μg/ml PmB at 37°C for 2 h or pretreated with 50 μg/ml proteinase K at 37°C for 1 h (Byun et al., 2012; Wen et al., 2014).

### Isolation of PEMs

Six- to eight-weeks-old C57BL/6 and TLR2/TLR4-deficient mice were injected intraperitoneally with 1 ml paroline. 4 days later, cells were recovered from euthanized mice by peritoneal lavage using 13 ml sterile PBS containing 5 mM EDTA. Cells were washed with Dulbecco's Modified Eagle Medium (DMEM) and seeded in tissue culture plates (5 × 10^5^/well for 24-well plate, 1 × 10^6^/well for 6-well plate). After 60 min, cells in tissue culture plates were washed with sterile PBS twice to remove non adherent cells. Recovered adherent cells were grown in DMEM supplemented with 1% penicillin-streptomycin and 10% fetal bovine serum (FBS) (HyClone, Barrington, USA) and stimulated as described below.

### Mouse model of intratracheal instillation of rPepO

TLR2/TLR4 deficient (TLR2KO/TLR4KO) mice were bought from The Jackson Laboratory (Bar Harbor, ME). Every mouse is on a C57BL/6 background. Experimental protocols involving animals were approved by the Animal Ethics and Research Committee of China University Graduate School of Medicine. Mice were 6–8 weeks old during experiments and were fed with standard rodent water and chow. All mice were not fasted prior to experiments. During experiments, each mouse was anesthetized with 80 μl pentobarbital. A mid ventral incision was performed. After isolation of muscles, the trachea was exposed. Then it was instilled with 50 μl rPepO (1 mg/ml) or 50 μl endotoxin-free PBS. At 24 h post instillation, every experimental mouse was humanely sacrificed to collect bronchoalveolar lavage fluid (BALF) or lung tissue, respectively. 2.5 ml sterile PBS was used to perform BALF.

### RNA extraction and PCR analysis

Total RNA was extracted from PEMs by RNAiso plus reagent (Takara) following manufacturer's instructions. Real-time PCR was performed in a Bio-Rad real time PCR machine with the use of SYBR Green detection protocol. All primers used were purchased from Sangon (Shanghai, China). Their sequences were listed below: β-actin, sense: 5′-TGCTGTCCCTGTA TGCCTCT-3′, antisense: 5′-GGTCTTTACGGATG TCAACG-3′; TNF-a, sense: 5′-GGCGGT GCCTATGTCTCA-3′, antisense: 5′-GGCAGCC TTGTCCCTTGA-3′; IL-6, sense: 5′-TGCCTT CTTGGGACTGAT-3′, antisense:5′-CTG GCTTTGTCTTTCTTGTT-3; CXCL1, sense: 5′-ACTCAAGAATGGTC GCGAGG-3′, antisense: 5′-GTGCCATCAGAGCA GTCTGT-3′; CXCL10, sense: 5′-CTGAGTCCTCGC TCAAGTGG-3′, antisense: 5′-CTAGGGAGGACAA GGAGGGT-3′; TLR2, sense: 5′-GAGCATCCGAATTGC ATCACC-3′, antisense: 5′-CCCAGAAGCATCA CATGACAGAG-3′; TLR4, sense: 5′- TTCAGAACT TCAGTGGCTGGATTTA-3′, antisense: 5′-GTCTCCACAG CCACCAGATTCTC-3′; After amplification protocol ended, Bio-Rad software was used for melt curve analysis of the PCR product. The gene for β-actin was used as an endogenous reference. Both relative delta delta Ct and a standard curve were used for determining quantification.

### Measurement of cytokines and chemokines

Cytokines and chemokines in BALF, lung homogenate, and cell culture medium were measured using ELISA kit for IL-6, TNF-α, and IL-17 (Biolegend), for CXCL10 (eBioscience), for CXCL1 (Biosepses), respectively, following the manufacture's instruction. In cell studies involving inhibitors, PEMs were pretreated with p38 inhibitor SB203580 (30 μM), ERK inhibitor U0126 (50 μM), JNK inhibitor SP600125 (40 μM), IκB-α phosphorylation inhibitor BAY11-7082 (20 μM), Janus kinase inhibitor AG490 (10 μM), or PI3K inhibitor LY294002 (20 μM) for 1 h at 37°C. The cells were then stimulated with rPepO for another 24 h at 37°C.

### Histology

Mice for histological analyses were anesthetized with lethal doses of pentobarbital followed by intratracheal infusion with 4% paraformaldehyde (PFA) at 24 h post instillation. The lung tissues were dissected and fixed (4% PFA) overnight. Serial 5-μm paraffin sections of lung tissues were stained by hematoxylin-eosin and observed with a light microscope (Liu et al., 2014). Cells in BALF from WT, TLR2-deficient or TLR4-deficient mice were diluted and stained with Wright-Giemsa dye.

### Flow cytometry analysis

Cells in BALF were collected and fixed with 4% PFA for 15 min. After washing with precooled PBS, cells were re-suspended with Fc Block (BD Pharmingen, San Diego, CA) at a dilution of 1:100 in PBS and incubated on ice for 60 min. Then the cells were probed in the dark with corresponding APC-conjugated anti CD11b (eBioscience), FITC- conjugated anti Ly6G (eBioscience), PE-conjugated anti F4/80 (eBioscience), or appropriate isotype control Ab (eBioscience) for 1 h on ice. Labeled cells were analyzed by a FACS calibrator counting 10,000 events per sample.

### Immunofluorescence assays

PEMs were seeded on poly-l-lysine -coated glass coverslips overnight. After stimulation with rPepO (10 μg/ml) for indicated time, cells on coverslips were fixed with 4% PFA for 15 min, permeabilized with 0.1% Triton X-100 for 5 min, and blocked with 2% BSA in PBS for 2 h. Then these cells were probed with anti-histidine antibody or anti-NF-κB P65 antibody for 2 h at room temperature. After washing in PBS, the cells were incubated with Alexa 594 or 488-conjugated secondary antibody in a dark room for 1 h and were then stained with DAPI for 5 min at room temperature. Cell morphology and fluorescence intensity were observed with a Nikon ECLIPSE 80i microscope equipped with a Nikon INTENSILIGHT C-HGFI.

### Western blot analysis

Various pneumococcal serotype isolates were grown in C+Y medium and collected. After washing with pre chilled PBS, PEMs were lysed with lysis buffer (RIPA containing phosphorylase inhibitor and protease inhibitor mixed with SDS loading buffer) for 15–30 min on ice and boiled for 5 min. Before transferring to a PVDF membrane (Millipore, Bedford, MA), the same volume of lysate (10 μg) was separated by 10% SDS–PAGE. After blocking with 5% defatted milk for 2 h at 37°C, the membrane was probed with indicated antibody at 4°C overnight. The membrane was washed for three times, and incubated with appropriate secondary goat anti-rabbit or goat anti-mouse antibodies for 1 h at 37°C. Then an ECL western blotting detection system was used to detect antibody–antigen complexes. Band intensity was quantified by Quantity one software (Bio-Rad, Hercules, CA) to determine protein expression.

### Statistical analysis

All data were shown as mean ± SD of triplicate samples. Differences between groups were determined by Student's *t*-test or ANOVA followed by Tukey's test with the use of GraphPad Prism 5 (GraphPad Software, La Jolla, CA, USA). For all experiments, statistical significance was considered as *P*-values < 0.05.

## Results

### Preparation of rPepO and experiments to exclude the contamination of LPS in rPepO preparation

**Figure 1:**
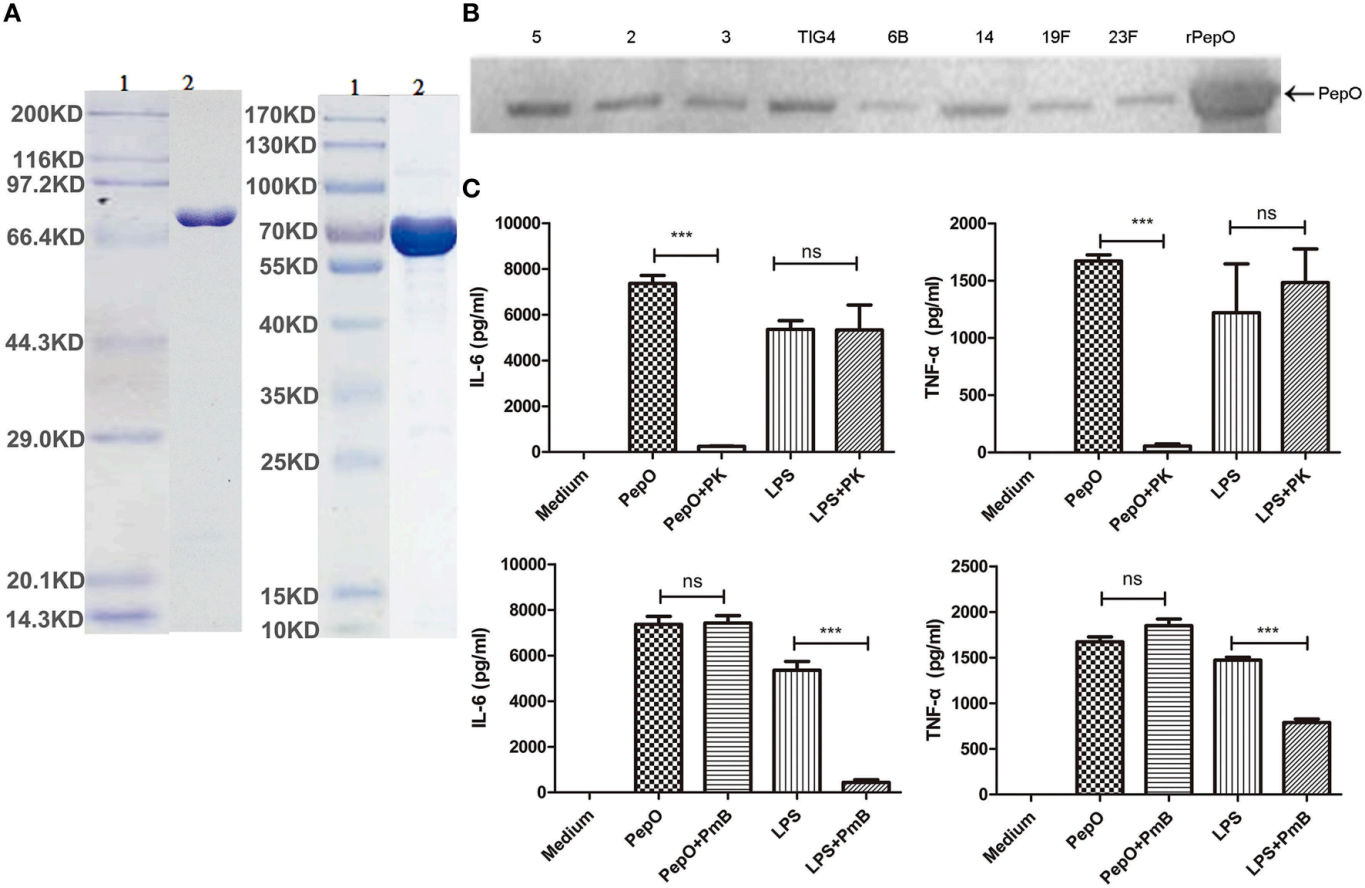
**Preparation of rPepO and experiments to exclude the contamination of LPS in rPepO preparation. (A)** rPepO (lane 2) was subjected to 10% SDS-PAGE and detected by Coomassie brilliant blue staining. Lane 1, protein marker or pre- stained marker. **(B)** Proteins from various pneumococcal serotype isolates were detected by anti-rPepO antibody using western bolt analysis. 5, 2, 3, TIG4, 6B, 14, 19F, 23F mean various pneumococcal serotypes. **(C)** rPepO (10 μg/ml) preparation and LPS (100 ng/ml) were digested with proteinase K (50 μg /ml) for 1 h or incubated with PmB (100 μg/ml) for 2 h at 37°C prior to adding to PEMs. TNF-α and IL-6 levels in culture medium were determined by ELISA. The data are shown as the mean ± SD (*n* = 3). Student's *t*-test was performed to calculate the statistical significance (^***^p < 0.001; ns, not significant).

The overexpressed rPepO was subjected to 10% SDS-PAGE and stained with coomassie blue. Figure 1A showed a single protein with the expected size, of which the purity was above 90%. Western blot analysis showed that the same size protein from various pneumococcal serotype isolates can be recognized by anti-rPepO antibody (Figure 1B), which further proves the correct expression of rPepO. LPS, a TLR4 agonist, is able to induce strong cytokine production (e.g., IL-6, TNF-α). Even though the final concentration of LPS in rPepO preparation was under 0.1 endotoxin units (EU)/ml, to avoid its effect, experiments to exclude the contamination of LPS were performed. PEMs stimulated with PmB-incubated rPepO secreted equal volume of IL-6 and TNF-α compared with cells stimulated with untreated rPepO. However, PEMs treated with protease K digested rPepO produced little IL-6 and TNF-α. At the same time, IL-6 and TNF-α induced by protease K digested LPS was equal to that induced by untreated LPS, while PmB-incubated LPS induced little release of IL-6 and TNF-α (Figure 1C). Taken together, these results suggested that the rPepO preparation contained too little LPS to influence our experiments.

### rPepO elicits strong innate immune response in lungs of *C57BL/6* WT mice

**Figure 2:**
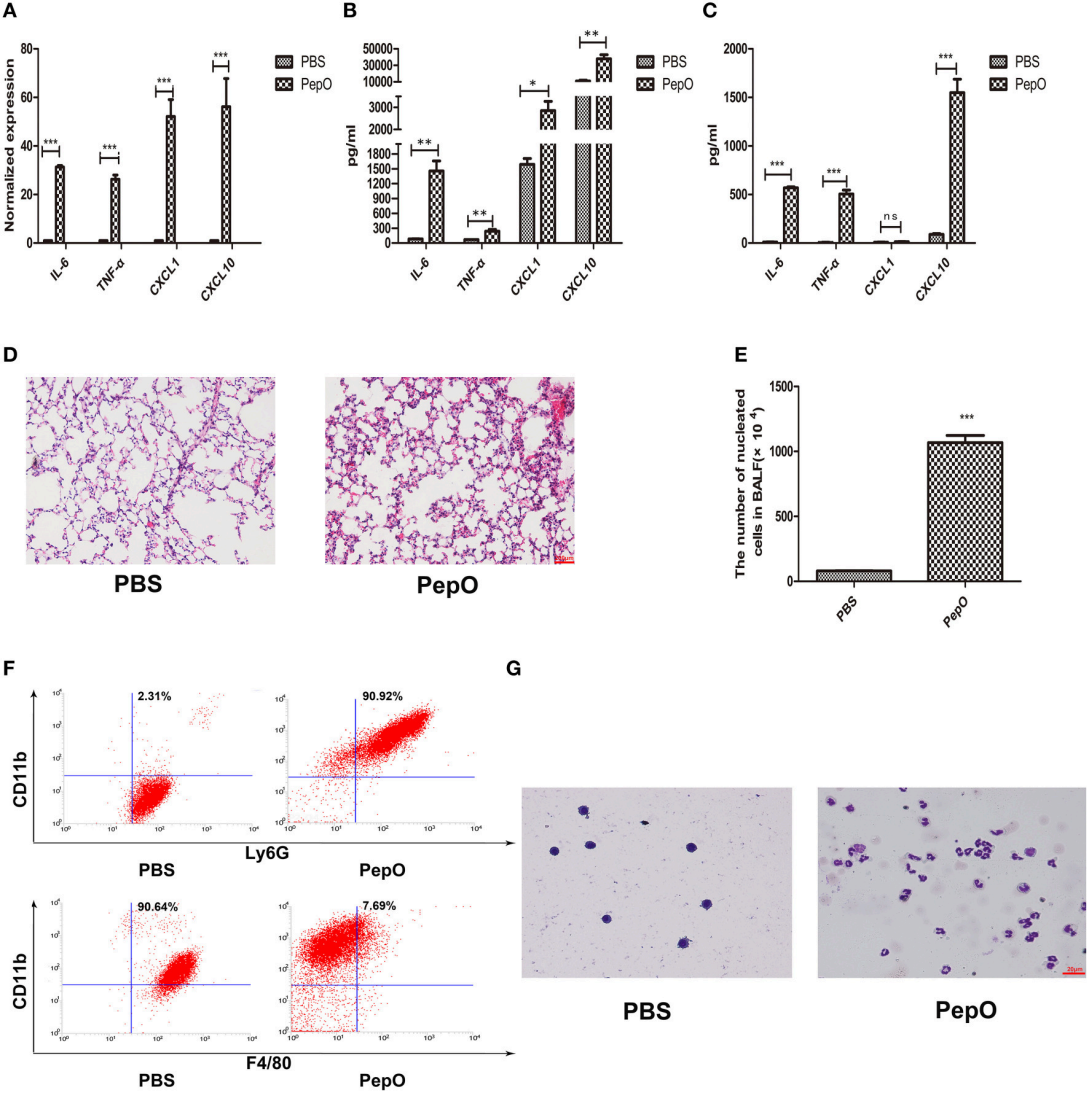
**rPepO elicits strong innate immune response in lungs of ***C57BL/6*** WT mice**. WT mice were intratracheally instilled with 50 μl rPepO (1 mg/ml) or 50 μl endotoxin-free PBS. The mice were humanely sacrificed at 24 h post instillation. **(A)** Total RNA was extracted from lung tissues and PCR analysis was used to assess the transcripts of cytokines. Cytokines production in Lung tissue **(B)** and BALF **(C)** were measured by ELISA. **(D)** Paraffin sections of the lung tissues were stained with hematoxylin –eosin (original magnification, × 20). **(E)** The number of nucleated cells in BALF was counted under light microscope. The cell types in BALF were analyzed by flow cytometry analysis **(F)** and Wright-Giemsa staining **(G)**. The data are shown as the mean ± SD (*n* = 3). Student's *t*-test was performed to calculate the statistical significance (^*^p < 0.05; ^**^p < 0.01; ^***^p < 0.001; ns, not significant).

To investigate the effect of rPepO on activation of host innate immunity, we constructed a mouse model of intratracheal instillation. *C57BL/6* WT mice were intratracheally instilled with rPepO or endotoxin-free PBS. 24 h later, IL-6, TNF-α, CXCL1, and CXCL10 transcripts in lung tissues of rPepO administrated mice were significantly up-regulated compared with that of PBS administrated mice (Figure 2A). Consistently, protein levels of IL-6, TNF-α, CXCL1, and CXCL10 in lung homogenate and BALF of rPepO challenged mice were higher than that of PBS challenged mice (Figures 2B,C). Histology analysis showed that a number of cells were recruited to alveolar lumen of rPepO treated mice (Figure 2D). As shown in Figure 2E, the number of nucleated cells in BALF of rPepO stimulated mice was much more than that of PBS stimulated mice, with a mean increase of 10-fold. Flow cytometry analysis and Wright-Giemsa staining analysis demonstrated that 90.92% recruited nucleated cells in BALF from rPepO treated mice were neutrophils, while 90.64% nucleated cells in BALF from PBS treated mice were macrophages (Figures 2F,G). These results indicated the significant activation of mouse innate immunity by rPepO.

### rPepO-induced innate immune response is partially dependent on TLR2 and TLR4

**Figure 3:**
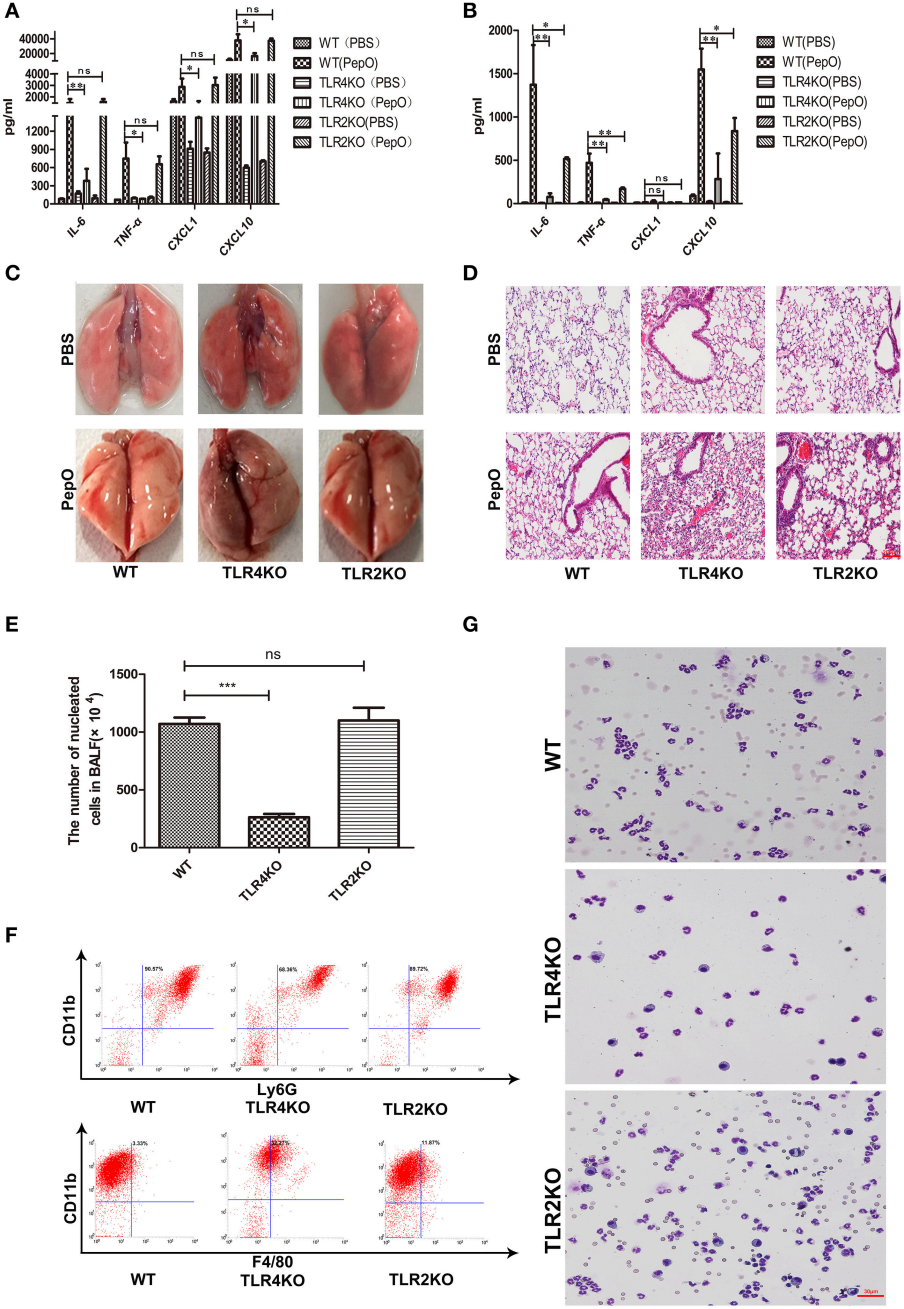
**rPepO– induced innate immune response is dependent on TLR2 and TLR4**. WT, TLR4 deficient and TLR2 deficient mice were intratracheally instilled with rPepO or PBS. 24h later, cytokines production (TNF-α, IL-6, CXCL1 and CXCL10) in lung homogenate **(A)** and in BALF **(B)** were measured by ELISA. **(C)** Pictures of lung tissues were taken. **(D)** Paraffin sections of the lung tissues were stained with hematoxylin –eosin (original magnification, × 20). **(E)** The number of nucleated cells in BALF of rPepO stimulated mice was counted under light microscope. The cell types in BALF were analyzed by flow cytometry analysis **(F)** and Wright-Giemsa staining **(G)**.The data are shown as the mean ± SD (*n* = 3). ANOVA followed by Tukey's test was performed to calculate the statistical significance (^*^p < 0.05; ^**^p < 0.01; ^***^p < 0.001; ns, not significant).

It has been proved that many pneumococcal proteins are sensed by TLR2 or TLR4, such as RrgA Pneumococcal Pilus Type 1 Protein, GHIP, Ply and so on (Malley et al., 2003; Basset et al., 2013; Dong et al., 2014). To investigate whether TLR2 and TLR4 participate in the recognition of rPepO, mouse models with TLR2 or TLR4 deficient mice were used. WT, TLR2 deficient and TLR4 deficient mice were intratracheally instilled with rPepO or endotoxin-free PBS. 24 h later, volume of TNF-α, IL-6 and CXCL10 in lung homogenate and BALF from rPepO stimulated TLR4 deficient mice was significantly less than that from rPepO stimulated WT mice. CXCL1 in lung homogenate, not in BALF of rPepO stimulated TLR4 deficient mice was reduced compared with that of rPepO stimulated WT mice (Figures 3A,B). Protein levels of IL-6, TNF-α, and CXCL10 in BALF from rPepO stimulated TLR2 deficient mice were obviously declined compared with that from rPepO stimulated WT mice, while in cytokines production of lung homogenate there was no significant difference between rPepO stimulated TLR2 deficient mice and WT mice (Figures 3A,B). Figures 3C,D showed that rPepO treated TLR2 or TLR4 deficient mice suffered intensified tissue injury. As shown in Figure 3E, the number of nucleated cells in BALF of rPepO administrated TLR4 deficient mice was significantly less than that of rPepO administrated WT mice, and there existed no significant difference between rPepO administrated TLR2 deficient mice and WT mice, which was consistent with the outcome of cytokines production in lung homogenate. Flow cytometry analysis and Wright-Giemsa staining analysis illustrated that the proportion of neutrophils in BALF of rPepO stimulated TLR4 deficient mice was declined, with a percentage of 68.36%. And there was no significant difference in proportion of neutrophils between rPepO stimulated TLR2 deficient mice and WT mice (Figures 3F,G). These results suggested that rPepO-induced innate immune response might play a protective role during pneumococcal infection and TLR4 may predominate in sensing rPepO. We were wondering how tissue injury in TLR2 and TLR4 deficient mice could occur without the inflammatory effects reported with WT mice. Other effects of rPepO such as fibrinogen binding or endopeptidase activity may participate in destroying the integrity of lung tissues.

### rPepO induces cytokines production in PEMs

**Figure 4:**
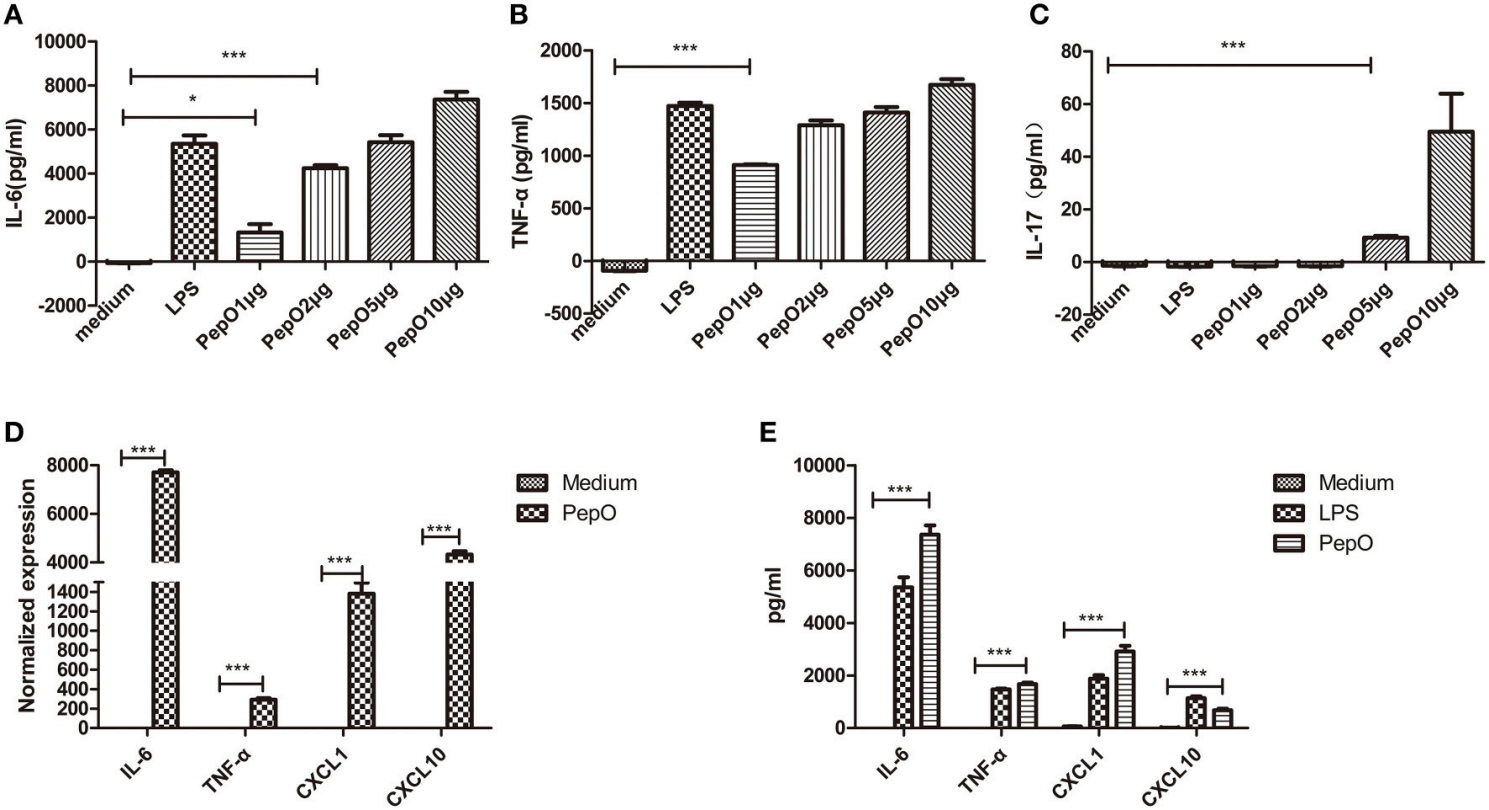
**rPepO induces cytokines production in PEMs. (A–C)** PEMs were treated with different dosages of rPepO or LPS (100 ng/ml) for 24 h, and IL-6, TNF-α, IL-17 in culture medium were determined by ELISA. **(D)** Total RNA was extracted from PEMs stimulated with rPepO (10 μg/ml) for 6 h and PCR analysis was used to measure the transcripts of cytokines. **(E)** Cytokines in culture medium of PEMs stimulated with rPepO (10 μg/ml) or LPS (100 ng/ml) for 24 h were measured by ELISA. The data are shown as the mean ± SD (*n* = 3). Student's *t*-test was performed to calculate the statistical significance (^*^p < 0.05; ^***^p < 0.001; ns, not significant).

Macrophages are key cells of the host innate immune system, so we focused our investigation on the effect of rPepO on macrophages *in vitro*. Primary PEMs were cultured and stimulated with different concentrations of rPepO. To be consistent with our mouse model we evaluated cells 24 h after rPepO treatment. 24 h later, viability of PEMs was above 95% (data not shown) as assessed by trypan blue exclusion staining, and secretion of IL-6, TNF-α, IL-17A increased in a dose-dependent manner (Figures 4A–C). And we chose concentration (10 μg/ml) of rPepO for subsequent experiments. After exposure to rPepO, release of IL-6, TNF-α, CXCL1, CXCL10 was dramatically augmented, both at the mRNA (Figure 4D) and protein level (Figure 4E).

### rPepO-induced cytokines production in PEMs is mediated by TLR2 and TLR4

**Figure 5:**
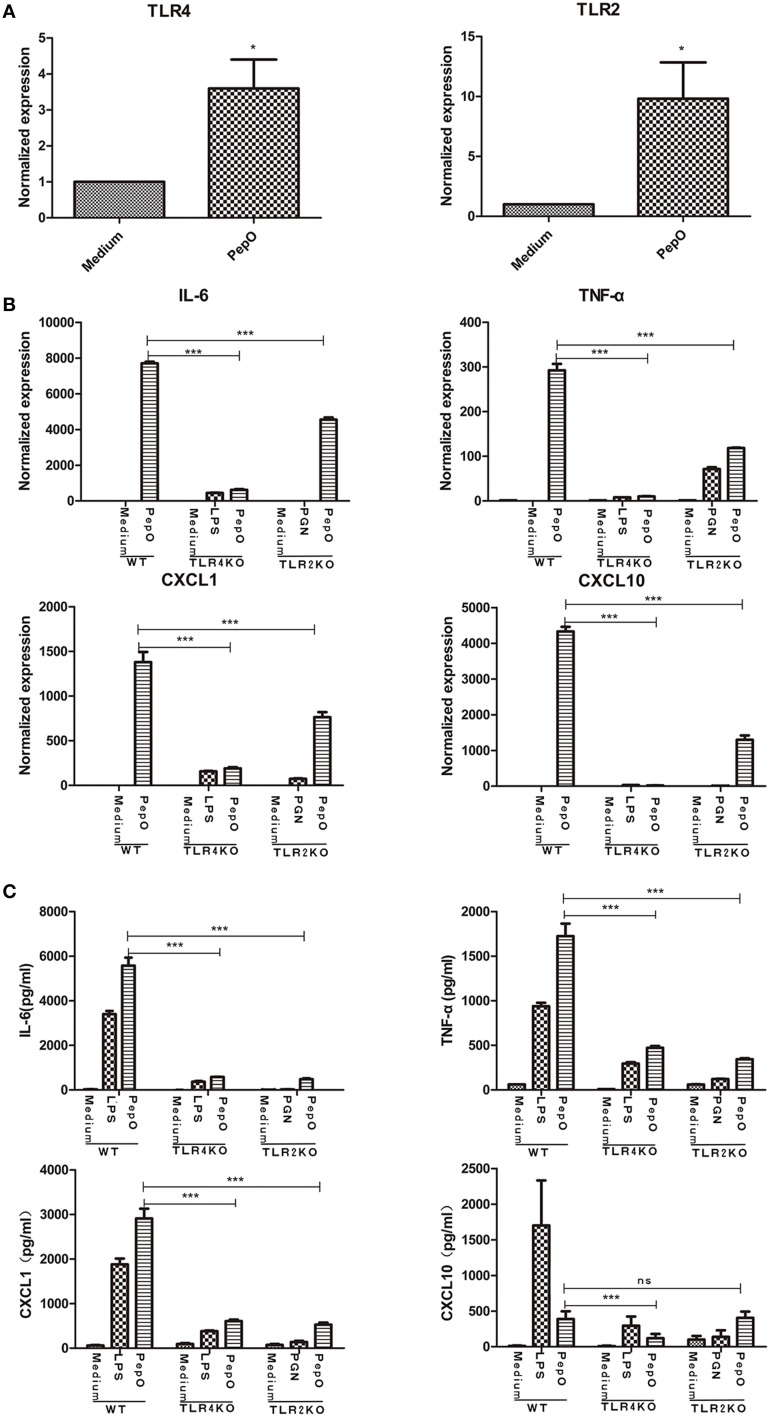
**rPepO - induced cytokines production in PEMs is mediated by TLR2 and TLR4. (A)** RNA was extracted from PEMs stimulated with rPepO (10 μg/ml) for 6 h and PCR analysis was used to determine TLR2 and TLR4 transcripts. **(B)** RNA was extracted from WT, TLR4, or TLR2 deficient cells stimulated by rPepO (10 μg/ml), LPS (100 ng/ml) or PGN (5 μg/ml) for 6 h and cytokines transcripts were evaluated by PCR analysis. **(C)** Cytokines in culture medium of WT, TLR2, or TLR4 deficient PEMs exposed to rPepO (10 μg/ml), LPS (100 ng/ml) or PGN (5 μg/ml) for 24 h were determined by ELISA. The data are shown as the mean ± SD (*n* = 3). Student's *t*-test was performed to calculate the statistical significance (^*^p < 0.05; ^***^p < 0.001; ns, not significant).

After exposure to rPepO, both TLR2 and TLR4 transcripts were up-regulated in PEMs (Figure 5A). Upon stimulation TLR2 or TLR4 deficient PEMs showed impaired secretion of cytokines and chemokines, both in mRNA and protein analyses (Figures 5B,C), suggesting that both TLR2 and TLR4 participated in the activation of rPepO on macrophages.

### rPepO induces phosphorylation of MAPKs, Akt and NF-κB P65 via TLR2 and TLR4 signaling

**Figure 6:**
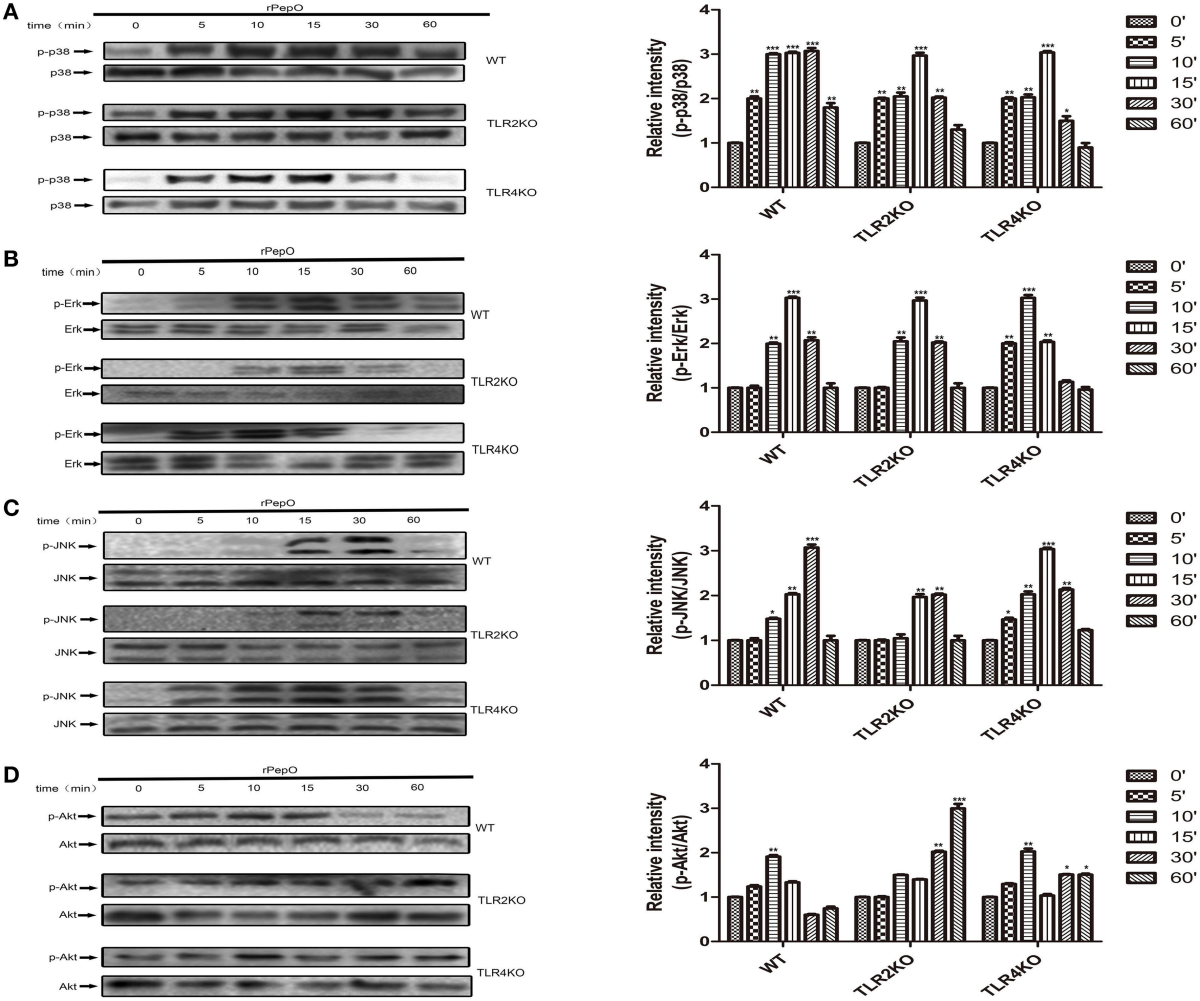
**rPepO induces phosphorylation of MAPKs and Akt via TLR2 and TLR4 signaling. (A–D)** WT, TLR2 or TLR4 deficient PEMs were incubated with rPepO (10 μg /ml) for the indicated times (0–60 min). Cell lysates were prepared, and western blot analysis was used to determine the phosphorylation of p38, Erk, JNK, and Akt. Representative blots of three independent experiments with consistent outcome are shown. The relative band intensities of three tests are shown in histograms above. The data are shown as the mean ± SD (*n* = 3). Student's *t*-test was performed to calculate the statistical significance (^*^p < 0.05; ^**^p < 0.01; ^***^p < 0.001).

**Figure 7:**
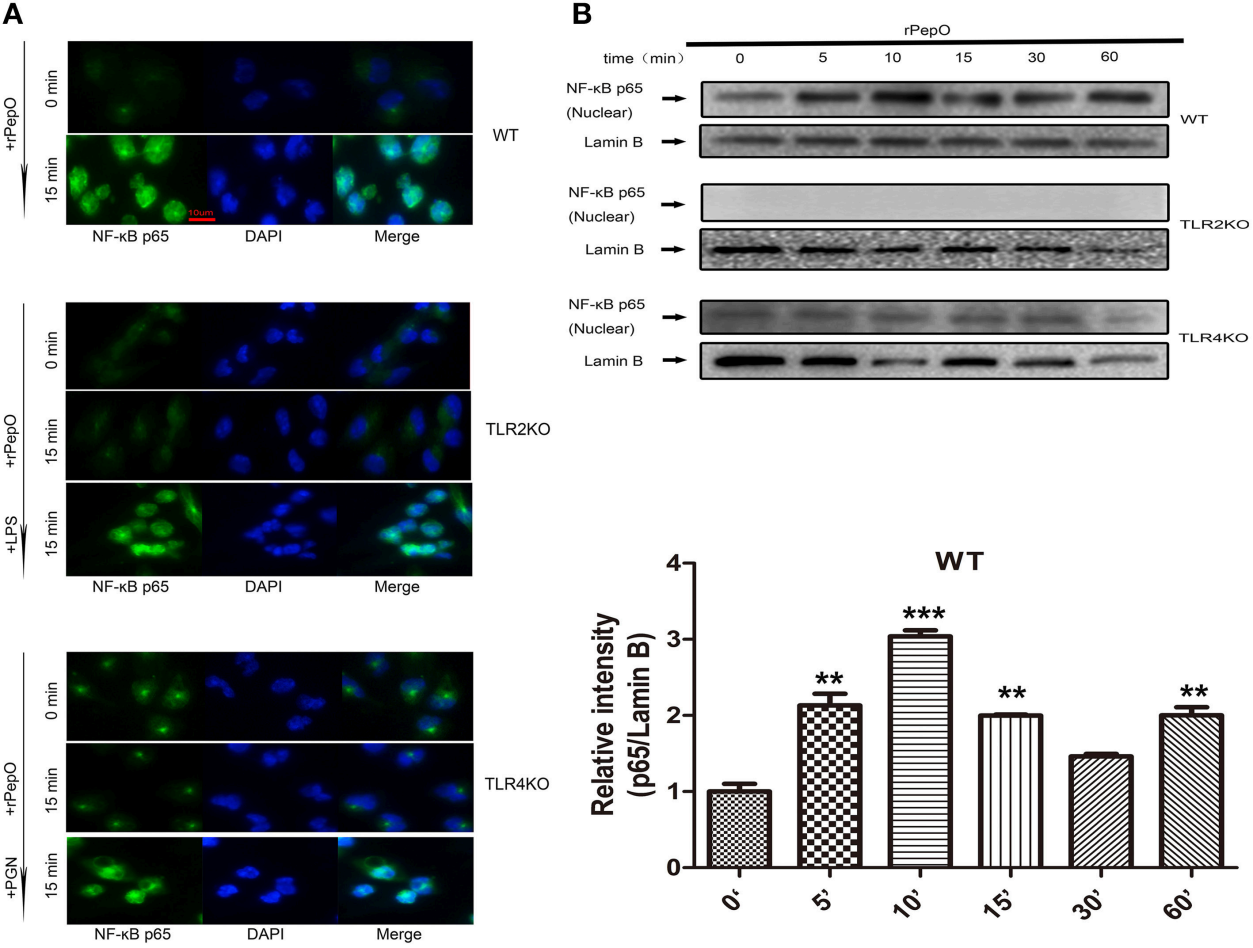
**rPepO induces nuclear translocation and phosphorylation of p65 in a TLR2 and TLR4 dependent manner**. Immunofluorescence **(A)** and western blot assays **(B)** were performed to determine the translocation and phosphorylation of NF-κB p65 in WT, TLR2 or TLR4 deficient PEMs. Representative blots of three independent experiments with consistent outcome are shown. The relative band intensities of three tests are shown in histograms above. Lamin B was used as a loading control for nuclear fractions. The data are shown as the mean ± SD (*n* = 3). Student's *t*-test was performed to calculate the statistical significance (^**^p < 0.01; ^***^p < 0.001).

The phosphorylation of MAPKs and Akt in PEMs stimulated by rPepO for different times were determined by western blot analysis. As shown in Figures 6A–D, rPepO induced a strong phosphorylation of MAPKs and Akt after 5–15 min stimulation, with a peak phosphorylation appearing at 10–15 min. In untreated cells little phosphorylated proteins were detected. The duration of p38 signaling response in TLR4 deficient PEMs was significantly less than that in WT or TLR2 deficient PEMs. While in TLR2 deficient cells activation of Akt was delayed but increased. Figure 7A showed the translocation of NF-κB p65 to nucleus of rPepO–treated WT PEMs. P65 was primarily present in the cytoplasm of unstimulated cells. While in TLR2 or TLR4 deficient cells, rPepO failed to induce the nuclear translocation of p65. Western blot analysis also demonstrated the phosphorylation of p65 in rPepO-stimulated WT cells (Figure 7B). While in TLR2 deficient cells, the phosphorylated p65 was undetectable. It is worth noting that p65 signaling analysis showed more ablated effects with TLR2 deficient cells than necessarily TLR4 deficient cells. Taken together, in TLR2 and TLR4 deficient cells the alteration of activation of intracellular signaling molecular may account for their impaired cytokines secretion.

### rPepO-induced cytokines production depends primarily on the activation of p38, Akt and NF-κB

**Figure 8:**
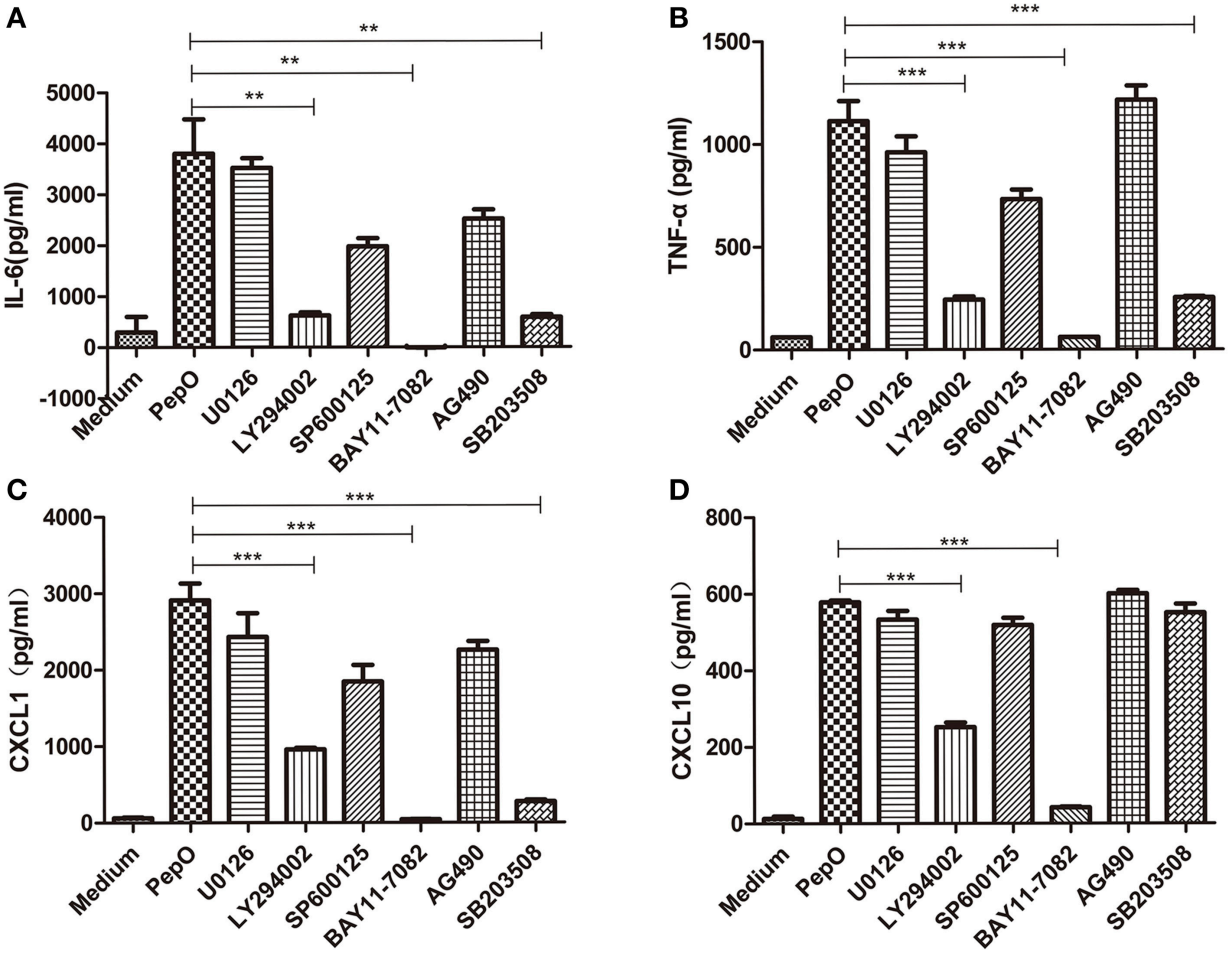
**rPepO-induced cytokines production depends primarily on the activation of p38, Akt and NF-κB. (A–D)** PEMs were pretreated with SP600125, U0126, SB203508, LY294002, AG490, and BAY11-7082 for 60 min, and incubated with rPepO (10 μg /ml) for another 24 hr. Cytokines production (TNF-α, IL-6, CXCL1, and CXCL10) were measured by ELISA. The data are shown as the mean ± SD (*n* = 3). Student's *t*-test was performed to calculate the statistical significance (^**^p < 0.01; ^***^p < 0.001).

PEMs were pretreated with appropriate amount of SB203580, U0126, SP600125, LY294002, AG490, or BAY11-7082 for 60 min, and stimulated by rPepO for another 24 h. TNF-α, IL-6, CXCL1 and CXCL10 in cell culture medium were measured by ELISA. Production of TNF-α, IL-6, CXCL1 was partially blocked by p38 inhibitor and PI3K inhibitor (50–70% inhibition), and significantly blocked by IκB-α phosphorylation inhibitor (above 90% inhibition) (Figures 8A–C). CXCL10 production was prominently blocked by NF-κB inhibitor and PI3K inhibitor (Figure 8D). Inhibitors of ERK, JNK, and JAK failed to influence the rPepO-induced cytokines production in PEMs (Figures 8A–D).

## Discussion

Recognition of microbial components by the innate immune system is an effective method to protect the host from various pathogens (Hoffmann et al., 1999). Pneumococcal PepO is a novel virulence factor, ubiquitously present in various pneumococcal isolates. Our results demonstrated that rPepO can activate host innate immune response against *S. pneumoniae*, inducing the production of TNF-α, IL-6, CXCL1, and CXCL10 both *in vivo* and *in vitro*. Cytokines play an important role in host defense against *S. pneumoniae*. TNF-α contributes to inhibiting the growth and transmission of pneumococci (van Der Poll et al., 1997a), which is mediated mainly by the TNF type I receptor (Kerr, 2002). Furthermore, during pneumococcal pneumonia TNF-α could compensate for the attenuated host defense response in type I IL-1 receptor-deficient mice (Rijneveld et al., 2001). IL-6 also protects host from pneumococcal infection through reducing the growth of pneumococci and prolonging survival (van Der Poll et al., 1997b). CXCL1 promotes neutrophils trafficking into lung tissue, which predominate the cellular infiltrates during pneumococcal pneumonia (Gauthier et al., 2007; Griffith et al., 2014). IL-17 was produced by rPepO stimulated PEMs, which also contributes to neutrophils trafficking (McAleer and Kolls, 2014). CXCL10 accelerates lymphocytes trafficking to dendritic cells (DCs) for priming and initiating adaptive immune response (Yoneyama et al., 2002; Griffith et al., 2014). In our model, INF-γ was not detectable (data not shown), maybe suggesting a protective role of rPepO-induced cytokines production, there is evidence showing that INF-γ inhibited antibacterial defense in models of either primary or post influenza pneumococcal pneumonia (Rijneveld et al., 2002; Sun and Metzger, 2008). Nevertheless, further research is still needed to identify the exact role played by the rPepO-induced cytokines production.

TLR2 and TLR4 are responsible for recognition of various kinds of microbial components. In the content of *S. pneumonia*, lipoproteins/lipopeptides, peptidoglycan, glycopolymers (lipoteichoic acid and lipoarabinomannan) and proteins (RrgA Pneumococcal Pilus Type 1 Protein, GHIP) have been shown to be TLR2 ligands (Paterson and Mitchell, 2006; Basset et al., 2013; Dong et al., 2014). Ply is a universally accepted agonist of TLR4. Our work illustrated that rPepO-induced cytokines production was mediated both in part by TLR2 and TLR4, and this is the first demonstration of both TLR2 and TLR4 mediated recognition of pneumococcal protein. Upon stimulation, TLR2 and TLR4 transcripts were up-regulated in WT PEMs. After treated with equal amount of rPepO, TLR2, or TLR4 deficient PEMs produced much less cytokines than WT PEMs did, both at mRNA and protein levels. In addition, in mouse model TLR4 deficient mice showed significantly decreased cytokines production, reduced neutrophils infiltration and intensified tissue injury compared with WT mice. While TLR2 deficient mice only showed decreased cytokines secretion in BALF accompanied by severer tissue injury. These results imply a protective role played by TLR2 and TLR4 in rPepO induced activation of host innate immunity. Previous study has proved that TRAM-dependent TLR4 signaling protect the host from endotoxic shock, which is controlled by p110δ of PI3K (Aksoy et al., 2012). In our model, whether rPepO induces a regulatory protective response via PI3K/Akt still needs further clarification. In mouse lungs rPepO –induced innate immune response is mainly mediated by TLR4. Other pulmonary cells may participate in rPepO-induced cytokines production via TLR4, which accounts for the disagreement of cytokines production between TLR2 deficient PEMs and mice. Previous study has shown that TLR4 signaling can induceTLR2 expression in endothelial cells (Fan et al., 2003). Whether crosstalk between TLR2 and TLR4 signaling occurs in rPepO-induced innate immunity still needs further exploration. We are also investigating the evidence of direct binding site of rPepO to TLR2 and TLR4.

Both TLR2 and TLR4 signaling pathways rely on TIRAP-MyD88- IRAK- TRAF6, which leads to the phosphorylation of NF-κB and MAPKs. Additionally TLR4 signaling can be also mediated by TRAM-TRIF adaptor complex (Akira and Takeda, 2004; Ashall et al., 2009). Our studies showed that rPepO induced activation of MAPKs, PI3K/Akt and NF-κB. These intracellular messenger molecules were phosphorylated rapidly in response to rPepO. There was no significant difference in activation of MAPKs and Akt among WT, TLR2 deficient and TLR4 deficient PEMs, implying that the rPepO-induced activation of innate immunity likely mediated by both TLR2 and TLR4. Either TLR2 or TLR4 signaling can result in the phosphorylation of MAPKs and Akt (Akira and Takeda, 2004). While rPepO failed to induce translocation of p65 to the nucleus and phosphorylation of p65 in TLR2 or TLR4 deficient PEMs, suggesting the non- redundant role of TLR2 and TLR4 played in rPepO-induced innate immune response. Furthermore, rPepO-induced cytokine production was partially mediated by p38/MAPK and PI3K/Akt signaling, and mainly mediated by NF-κB p65 signaling. Cytokine production was partially blocked by p38 inhibitor and PI3K inhibitor (50–70% inhibition), but almost completely blocked by IκB-α phosphorylation inhibitor (above 90% inhibition). Compared with WT PEMs, rPepO– treated TLR2 deficient cells showed delayed activation of Akt and abolished phosphorylation of p65, while TLR4 deficient cells showed shorter duration of p38 signaling and ablated activation of p65, which may account for their impaired cytokines production.

It is worth noting that an equal amount of rPepO failed to activate NF-κB p65 in TLR2 or TLR4-deficient PEMs. NF-κB regulates responses to pathogens and stresses in many mammalian cell types and it is the main transcription factor phosphorylated in TLRs signaling. The dimeric transcription factor is composed of five Rel family proteins (Ashall et al., 2009). Among these NF-κB proteins only p50 and p65 (also known as RelA) are readily detectable in lung nuclear fractions during acute pulmonary inflammation (Blackwell et al., 1999; Mizgerd et al., 2002; Jones et al., 2005). P65 promotes the expression of inflammatory cytokines to drive inflammatory responses, and p65 deficient mice suffers severely impaired antibacterial defense (Quinton et al., 2007). Besides, in mouse models of pneumococcal infection inhibition of NF-κB activation helps with bacterial growth and increases lethality (Quinton et al., 2007). In the present study, the fast activation of p65 may play a protective role in rPepO-induced innate immune response in PEMs. The failure of activation of P65 in TLR2 and TLR4 deficient cells could in some extent account for the intensified tissue injury in TLR2 and TLR4 deficient mice.

Previous researches have demonstrated that TLR activation in B cells is an important mediator of antibody responses against certain adjuvant vaccines (Pasare and Medzhitov, 2005; Kasturi et al., 2011). Consistently, the notion is supported by the recent data that flagellin ligands to TLR5 to act directly on activated B cells (Oh et al., 2014). As a TLR4 agonist, pneumococcal Ply has been proved to be a potent vaccine adjuvant (Douce et al., 2010). rPepO is a TLR2 and TLR4 agonist, whether it can be a vaccine adjuvant candidate still needs further investigation. We have demonstrated that rPepO stimulates the release of CXCL10, a TRIF-dependent type I interferon-inducible chemokine, which proves the activation of TRIF-dependent signaling in rPepO-induced innate immune response. And TRIF-mediated signaling results in less toxic immune stimulatory responses which is beneficial in boosting vaccine responses (Bordon, 2014; Kolb et al., 2014). So rPepO could be a vaccine adjuvant candidate.

Overall, our work has demonstrated for the first time the roles of a newer pneumococcal virulent factor, PepO, in activation of host innate immunity. We prove that rPepO induces strong host defense responses both *in vivo* and *in vitro* partially through the TLR2 and TLR4-signaling pathways. We also clarify the related molecular mechanism involved in the process.

## Author contributions

HZ, LK, XZ, and YY designed experiments; HZ, HY, ZS, and LK carried out experiments; HZ, LK, JH, and WX analyzed experimental results. HZ, LK, XZ, and XW wrote the manuscript.

### Conflict of interest statement

The authors declare that the research was conducted in the absence of any commercial or financial relationships that could be construed as a potential conflict of interest.
